# The protective effect of 999 XiaoErGanMao granules on the lungs and intestines of influenza A virus-infected mice

**DOI:** 10.1080/13880209.2023.2195884

**Published:** 2023-04-10

**Authors:** Yuan-zhen Hao, Li-feng Cen, Ting Wang, Tong Yi, Xun-long Shi, Hui-juan Duan, Zhi Dai, Hai-yan Zhu, Jian-guo Tang

**Affiliations:** a Department of Biological Medicines & Shanghai Engineering Research Center of ImmunoTherapeutics, School of Pharmacy, Fudan University, Shanghai, China; b China Resources Sanjiu Medical & Pharmaceutical Co., Ltd, Shenzhen, China; c Department of Trauma-Emergency & Critical Care Medicine, Shanghai Fifth People’s Hospital, Fudan University, Shanghai, China

**Keywords:** Influenza virus, viral pneumonia, intestines injury

## Abstract

**Context:**

Gastrointestinal symptoms are a common complication of influenza virus infection in children, which the gut-lung axis become involved in its biological progress. The protective effect of 999 XiaoErGanMao granules (XEGMG) on multi-organ injury in viral pneumonia remains unclear.

**Objective:**

To investigate the therapeutic effect of XEGMG on lungs and intestines injury in A/FM/1/47 (H1N1) influenza virus-infected mice.

**Materials and methods:**

Male BALB/c mice were infected with the 2LD_50_ H1N1 influenza virus and then treated with XEGMG (6 or 12 g/kg) intragastrically once a day for 4 days. The lung and colon samples were then collected for pathological observation, and assays for inflammatory cytokines and intestinal barrier. Mouse feces were collected to evaluate the intestinal microbiota.

**Results:**

Treating with XEGMG (12 g/kg) can mitigate body weight loss caused by 2LD_50_ H1N1 infection. It can also reduce lung index and pathological damage with the decreased inflammatory cytokines such as IL-6 and IL-1β. Furthermore, XEGMG (12 g/kg) can maintain the goblet cell number in the colons to protect the intestinal barrier and regulate the major flora such as Firmicutes, Bacteroidetes, and Muribaculaceae back to normal. Meanwhile, the expression of IL-17A in the colon tissues was significantly lower in the group of XEGMG (6, 12 g/kg) compared to H1N1 group.

**Discussion and conclusions:**

XEGMG can protect against H1N1 invasion involved in gut-lung axis regulation. The results provide new evidence for the protective effect of XEGMG, which is beneficial to vulnerable children.

## Introduction

Influenza virus infection is an acute infectious respiratory disease that seriously threatens human health (Iuliano et al. 2018; Krammer et al. 2018). Influenza A virus (IAV) is a common cause of pediatric respiratory diseases (Kumar 2017). The lungs, intestines, spleens, and other organs of children are not fully developed and hence more vulnerable to the influenza virus, as per the consensus reached based on the theory of Traditional Chinese Medicine (TCM). Past research has demonstrated that there have been 109.5 million influenza virus episodes in children under 5 years across the world in 2018 alone (Wang et al. 2020). Common symptoms after influenza infection include cough, fever, and headache, which are the same as those in adults (Fraaij and Heikkinen 2011; Ruf and Knuf 2014; Nayak et al. 2021; Watanabe et al. 2021). Influenza virus infection often causes viral pneumonia in the face of weak immune defense and excessive viral burden in children (Wang et al. 2020). Notably, in children, viral pneumonia is often accompanied by gastrointestinal symptoms, such as nausea, vomiting, abdominal pain, and diarrhea (Wang et al. 2003; Hien et al. 2004; Abdel-Ghafar et al. 2008), implying that IAV can indirectly induce an intestinal immune injury.

The gut-lung axis has been confirmed to be involved in the pathological process of lung and intestinal injury induced by the influenza virus. The intestinal tract is extremely sensitive to ischemia and hypoxia-induced by viral pneumonia, leading to intestinal barrier damage and intestinal homeostasis disorder. The gut is the largest organ vulnerable to microbial colonization. The gut microbiota can modulate not only local immunity but also distant immune responses (Round and Mazmanian 2009; Thaiss et al. 2016). The normal intestinal flora participates in the digestion, absorption, and metabolism of the host, thereby constituting a biological barrier to prevent the invasion of harmful substances (Kamada et al. 2013; Pickard et al. 2017; Caruso et al. 2020; Lo et al. 2021). The diversity and stability of the gut microbiota can thus alleviate viral lung injury (Ichinohe et al. 2011). Evidently, the disturbances in the gut microbiota can affect not only the inflammation in the gut but also that in the respiratory tract (Wang et al. 2014). Animal experiments have demonstrated that gut bacteria can resist bacterial and viral pneumonia by modulating innate and adaptive immune responses (Ichinohe et al. 2011; Chen et al. 2017; Sencio et al. 2022; Yang et al. 2022). Understandably, adverse effects can occur when the intestinal barrier gets impaired. Moreover, the composition of the gut microbiota, which can be altered by the diet, medication, or disease, plays an important role in pulmonary immune responses and immune homeostasis in the respiratory tract (Clarke et al. 2014; Bressa et al. 2017).

Antiviral drugs can help shorten the course of the disease and prevent serious complications. The Centers for Disease Control and Prevention have recommended four Food and Drug Administration–approved antiviral drugs for the treatment of influenza (CDC 2022): oseltamivir (oral), baloxavir (oral), zanamivir (inhaled), and peramivir (intravenous). The NA inhibitor oseltamivir is a recognized anti-influenza drug at present, albeit its wide application is limited due to the short treatment time window and the gastrointestinal side effects. However, 999 XiaoErGanMao granules (XEGMG) are a common clinical medicine used to treat children’s colds in China. In this study, XEGMG was provided by China Resources Sanjiu Medical & Pharmaceutical Co., Ltd. In the past 5 years, the average annual sales of XEGMG were approximately 66.26 million RMB (MINEI 2022). Considering its beneficial effect, XEGMG has been the first choice of over-the-counter (OTC) drug for several children with colds in China. XEGMG has a complex constitution that contains 10 TCM components, including *Pogostemon cablin* Benth. (Lamiaceae), *Chrysanthemum morifolium* Ramat. (Asteraceae), *Forsythia suspensa* Vahl (Oleaceae), *Isatis indigotica* Fort. (Brassicaseae), *Isatis tinctoria* L. (Brassicaseae), *Rehmannia glutinosa* Libosch. (Scrophulariaceae), Cortex Lycii (Solanaceae), *Cynanchum atratum* Bge. (Asclepiadaceae), *Mentha canadensis* Linnaeus (Lamiaceae), and Gypsum Fibrosuum. The classic theory of children’s bodies is characterized by the deficiency of the spleens and lungs presented in TCM pediatrics, which has been helpful to comprehend the susceptibility to influenza virus infection. Therefore, an effective pediatric drug that can alleviate both lung and intestinal immune injury seems more promising for children’s recovery. Our past studies have confirmed that influenza simultaneously induces lung and intestinal damage, and polysaccharides derived from plants can effectively protect the lungs and intestines by regulating the balance of Th17/Treg cells in the gut-lung axis, which has provided the foundation for this subject (Zhu et al. 2018; Shi et al. 2020). The immune regulatory mechanisms of XEGMG, however, remain unclear. Through this study, we elucidate the protective effect of XEGMG on the lungs and intestines of IAV-infected mice, which provides evidence for its efficiency and superiority in clinical application.

## Materials and methods

### Drug preparation

The drugs used in this study included OST (Roche, Shanghai, China, J20140121) and XEGMG (China Resources Sanjiu Medical & Pharmaceutical Co., Ltd., Shenzhen, China). The drugs were weighed before use and then triturated with sterile warm water to achieve the desired concentration. All prepared drugs were stored at 4 °C and sonicated before use.

### Animals and ethics statement

Male BALB/c mice (age: 4–6 weeks, weight: 14–16 g) were obtained from the Slaccas-Shanghai Lab Animal Ltd. (SPF II; Certificate No. SCXK2012-0002). The mice were housed in collective individually ventilated cages (IVCs) with free access to food and water. The rearing temperature was maintained at 20–25 °C and the humidity was set to 40–70%. All mice received humane care in accordance with the ‘Guide for the Care and Use of Laboratory Animals’ published by the National Institutes of Health. All study protocols were approved by the Animal Ethical Committee of the School of Pharmacy, Fudan University (Approval No. 2013-50).

### Influenza virus

The IAV/FM/1/47 (H1N1) was supplied by the Shanghai Centre for Disease Control & Prevention (Shanghai, China). H1N1 virulence was determined based on its median lethal dose (LD_50_) of 10^−4.3^. The virus was stored in aliquots at −80 °C. The influenza virus was diluted in RPMI 1640 medium (Meilunbio, Dalian, China) until further use.

### H1N1-induced acute lung injury and XEGMG administration

BALB/c mice were anesthetized with isoflurane by using a gas anesthesia apparatus (Mingweb, Zhongshan, China). The animals were infected with 2LD_50_ H1N1, except for group N. The mice were assigned to 5 groups of 6 each, as follows: N (negative control, infected with the RPMI 1640 medium), H1N1 (infected with H1N1 alone), 999 L (infected with H1N1 and intragastrically treated with 6 g/kg XEGMG), 999H (infected with H1N1 and treated intragastrically with12 g/kg XEGMG), OST (infected with H1N1 and treated intragastrically with 22.75 mg/kg oseltamivir). Changes in body weight were recorded daily after the mice were infected.

### Enzyme-linked immunosorbent assay (ELISA)

Four samples from each group were prepared for the lung and colon homogenates in PBS at the concentration of 100 mg tissue/mL using the Tissue Grinder (Jingxin, Shanghai, China) at 50 HZ for 1 min. The total protein content in the homogenates was detected by using the BCA Kit (Beyotime, Nantong, China). The expression of interleukin-17A (IL-17A) in the colon homogenate and that of interleukin-1β (IL-1β), interleukin-6 (IL-6) in the lung homogenates were assessed by ELISA kits individually (Abclonal, Shanghai, China) and detected with a microplate reader (Epoch, BioTek, USA). All assays were performed in accordance with the manufacturer’s instructions.

### Hematoxylin–eosin (H&E) staining

The colon and the upper lobe of the left lungs of the mice were fixed with 4% paraformaldehyde. After embedding in conventional paraffin, the tissue was serially sectioned at 4 μm. After dewaxing and hydration, the sections were sequentially stained with hematoxylin solution and eosin solution. The pathological changes in the slices were observed under a light microscope.

### Periodic acid–Schiff (PAS) staining

The sections were prepared as described for H&E staining. After dewaxing and hydration, these sections were sequentially incubated in the periodic acid solution, Schiff reagent, and hematoxylin solution in turn in accordance with the manufacturer’s instructions (Catalog No. BA-4080A, Baso Biotechnology Co. Ltd., Zhuhai, China). The pathological changes were determined and recorded under a light microscope.

### 16S rRNA gene amplicon analysis

The DNA was extracted from the fecal samples and its concentration was determined. Using 20–30 ng DNA as a template, 16S rDNA was amplified with PCR primers, and a linker with an index was added to the end of the PCR product of 16S rDNA for Next-Generation Sequencing (NGS). After the purification of the library with magnetic beads, the concentration was detected with a microplate reader, and the fragment size was determined by agarose gel electrophoresis. Then, the sequence information was read with the Illumina MiSeq/Novaseq instrument.

### Statistical analysis

All statistical analyses were performed using the GraphPad Prism for Windows (Version 9.0). Experimental data were evaluated by Student’s *t*-test (comparison of two groups). In all cases, *p*** **<** **0.05 was considered to indicate statistical significance.

## Results

### XEGMG protected against pulmonary injury in IAV-infected mice

**Figure 1.**
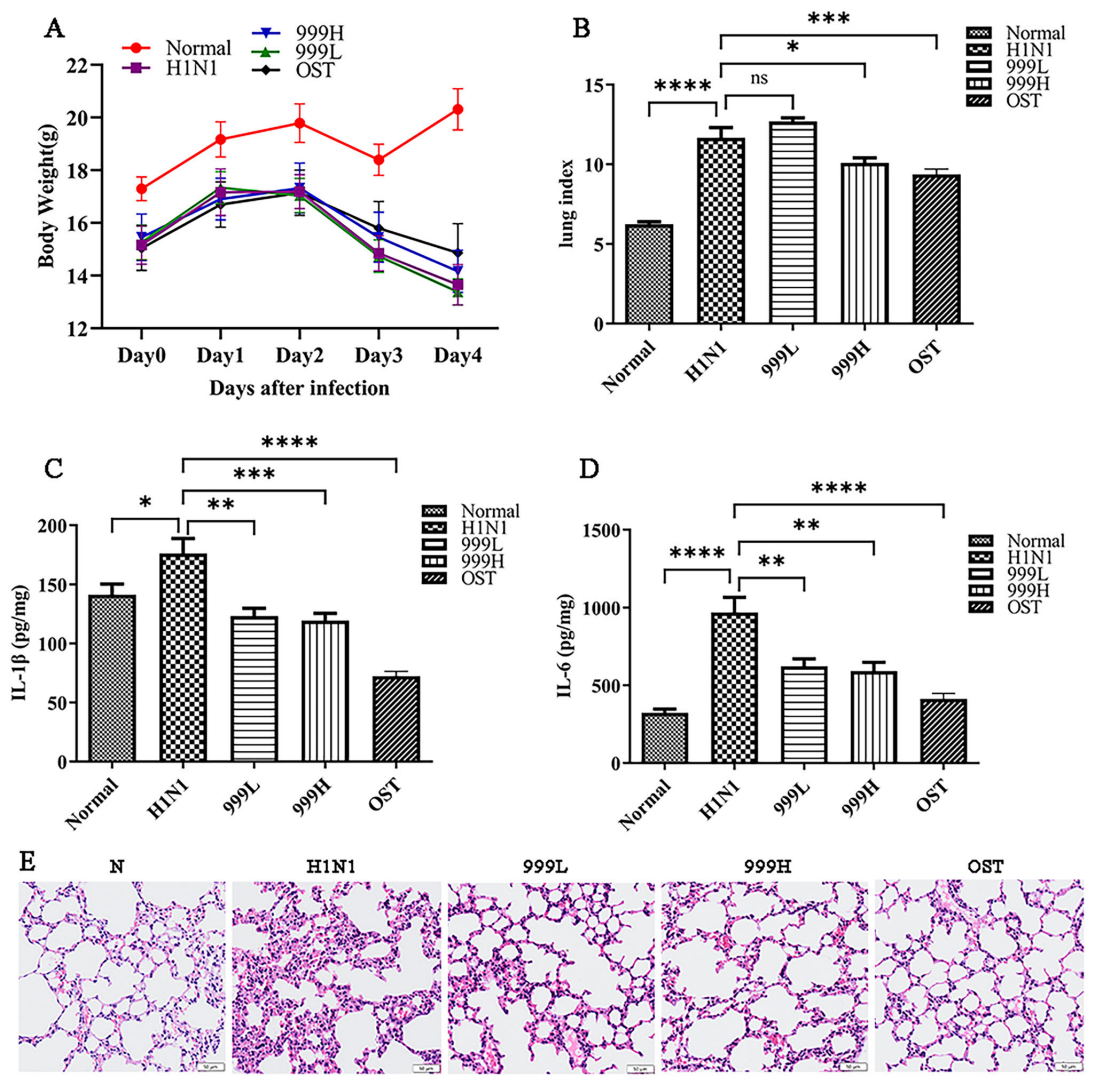
XEGMG demonstrated protective effects against pulmonary injury in IAV-infected mice. The mice were infected intranasally with the influenza virus at the dose of 2 LD_50_/mouse in 30 μL of the RPMI 1640 medium and administered 2 h after infection at day 0 (*n* = 6). (A) We monitored changes in the body weight for 5 days. (B) Lung indices of the infected mice were analyzed on day 4 after the H1N1 infection. Lung index = lung weight of mice (mg)/body weight of mice (g) (*n* = 6). (C, D) The expression of inflammatory cytokines IL-1β and IL-6 were measured by ELISA on day 4 in the lung homogenates of the infected mice (*n* = 4). (E) Representative histological H&E staining images of the lung on day 4 (Scale bar: 50 μm). Data are presented as the mean ± standard error of the mean (*p < 0.05; **p < 0.01; ***p < 0.001; ****p < 0.0001 when compared with the H1N1 group). H1N1, infected with A/FM/1/47(H1N1); LD_50_, median lethal dose; N, mock-infected control group; 999 L, the infected mice were treated with 6 g/kg XEGMG intragastrically; 999H, treated with 12 g/kg XEGMG intragastrically; OST, treated with 22.75 mg/kg oseltamivir intragastrically; IL, interleukin; H&E staining, Hematoxylin–Eosin staining.

To determine whether XEGMG could prevent IAV-induced lung injury, BALB/c mice were infected with 2LD_50_ of H1N1. The results revealed that H1N1 infection led to weight loss (Figure 1(A)) along with an increased lung index (*p*** **<** **0.001) from pulmonary congestion and edema, indicating that H1N1 infection can cause lung tissue swelling. In addition, high-dose XEGMG (12 g/kg) treatment was found to alleviate lung injury which was indicated by the significance of lung index compared with the H1N1 group (*p*** **<** **0.05) (Figure 1(B)). Meanwhile, to evaluate the secretion level of inflammatory mediators, we determined the expression of IL-1β and IL-6 in the lung homogenate, and the results implied that when compared with the negative control group, the expression of IL-1β (*p*** **<** **0.05) and IL-6 (*p*** **<** **0.0001) in the mice were significantly increased after H1N1 infection, and they were restored after treatment with XEGMG (*p*** **<** **0.01) (Figure 1(C,D)). In addition, the morphological observation of lung histopathology revealed that most H1N1-infected mice exhibited severe monocyte and lymphocyte infiltration, thickening of the alveolar walls, and infiltration of the inflammatory cells into the alveolar space (Figure 1(E)). When compared with the H1N1 group, XEGMG (12 g/kg) treatment significantly alleviated lung lesions, and inhibited the infiltration of monocytes and lymphocytes, which led to a reduction in inflammation. These results together indicated that high doses of XEGMG could prevent IAV-induced lung injury.

### XEGMG improved gut injury in IAV-infected mice

**Figure 2.**
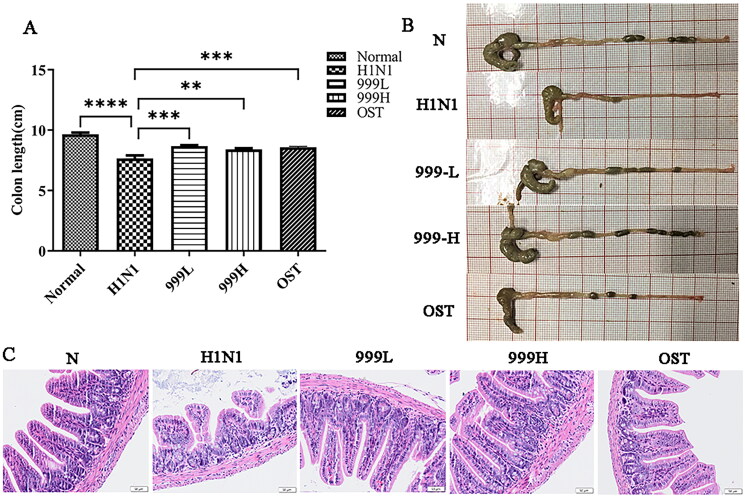
XEGMG improved gut injury in IAV-infected mice. The mice were subjected to colon-related analysis after 4 consecutive days of dosing after infection. (A) The length of the colons at day 4 (*n* = 5). (B) The representative images of the colons at day 4. (C) Representative histological H&E staining images of the colons at day 4 (Scale bar: 50 μm). Data are presented as mean ± standard error of the mean (*p < 0.05; **p < 0.01; ***p < 0.001; ****p < 0.0001 when compared with the H1N1 group). H1N1, infected with A/FM/1/47(H1N1); N, mock-infected control group; 999 L, the infected mice were treated with 6 g/kg XEGMG intragastrically; 999H, treated with 12 g/kg XEGMG intragastrically; OST, treated with 22.75 mg/kg oseltamivir intragastrically; H&E staining, Hematoxylin–Eosin staining.

Influenza virus can cause indirect immune damage to the intestine. As shown in Figures 2(A,B), the colons of the H1N1 group were found to be significantly shorter than those of the negative control group (*p*** **<** **0.0001). Treating with XEGMG significantly increased the length of colons relative to that in the H1N1 group (*p*** **<** **0.01). The results of the pathological staining of the colon tissues are shown in Figure 2(C). The negative control mice had a normal colonic tissue structure, complete mucosal layers, regular gland arrangement, and no infiltration of the inflammatory cells. However, the results of the model group demonstrated that H1N1 infection induced significant ulceration in the colon tissues of mice, severe mucosal damage, disordered glandular structure, and excessive inflammatory cell infiltration. When compared with the H1N1 group, both high and low doses of XEGMG were found to maintain the integrity of the intestinal mucosa, showcased uniform gland arrangement, and displayed a reduced number of infiltrated inflammatory cells. These results suggested that the use of XEGMG (12 g/kg) has significant efficiency on the gut, not limited to the lungs.

### XEGMG exhibited protective effects on the intestinal barrier of IAV-infected mice

**Figure 3.**
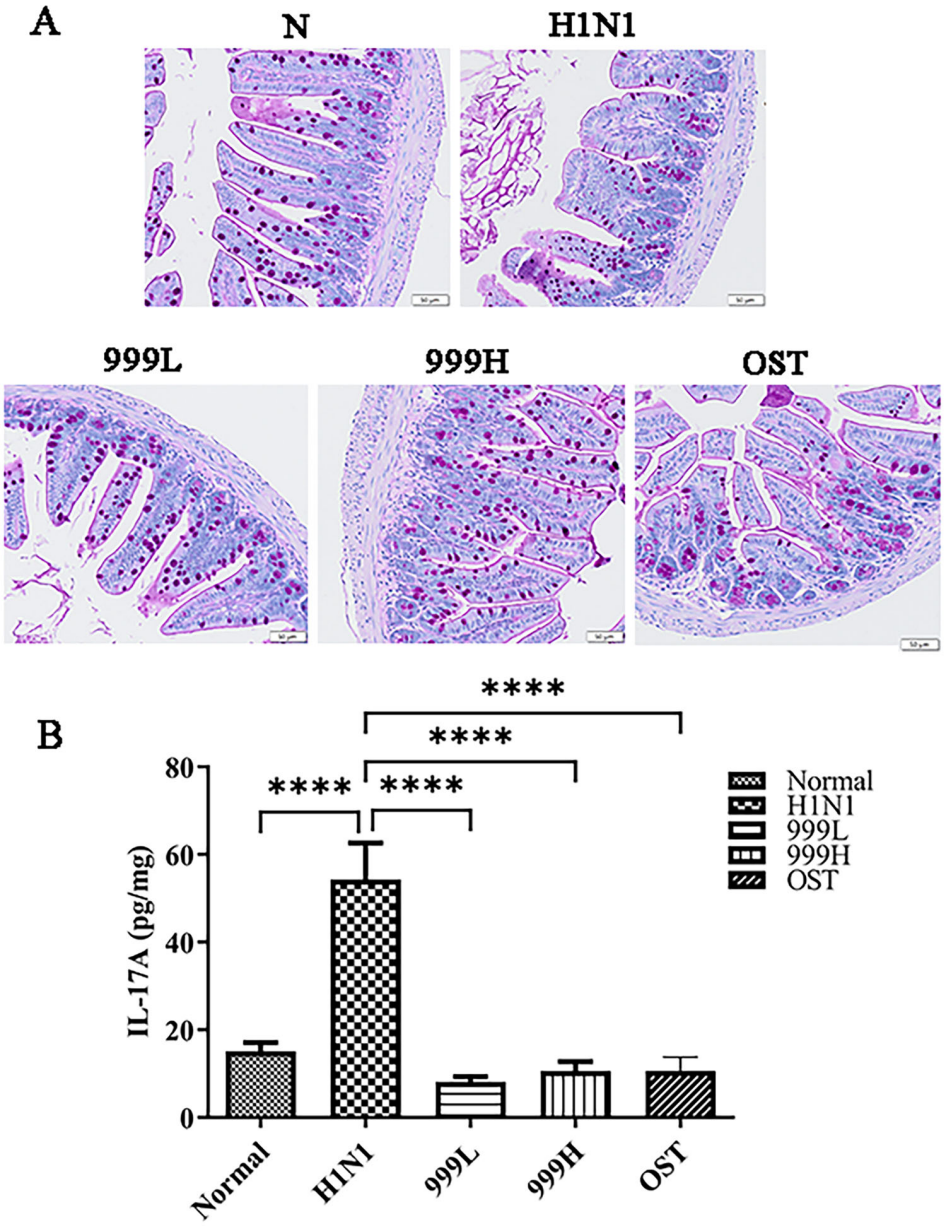
XEGMG demonstrated protective effects on the intestinal barrier of IAV-infected mice. (A) Representative histological PAS staining images of the colon at day 4 (Scale bar: 50 μm). (B) The expression of inflammatory cytokines IL-17A was measured by ELISA on day 4 in the colon homogenates of the infected mice (*n* = 4). Data are presented as the mean ± standard error of the mean (*p < 0.05; **p < 0.01; ***p < 0.001; ****p < 0.0001 when compared with the H1N1 group). H1N1, infected with A/FM/1/47(H1N1); N, mock-infected control group; 999 L, the infected mice were treated with 6 g/kg XEGMG intragastrically; 999H, treated with 12 g/kg XEGMG intragastrically; OST, treated with 22.75 mg/kg oseltamivir intragastrically; PAS staining, Periodic Acid–Schiff (PAS) staining; IL, interleukin.

Goblet cells are important components of the intestinal epithelial cells, which play a significant role in the gut’s innate immune system. The mucin, mucopolysaccharides, and other components secreted by the colonic goblet cells constitute a protective mucus barrier covering the intestinal surface (Deplancke and Gaskins 2001; Birchenough et al. 2015; Ma et al. 2018; Zhang and Wu 2020). Past studies have reported that the enterocytes undergo apoptosis after rotavirus infection in mice. It can also induce significant changes in goblet cell abundance, function, and differentiation (Boshuizen et al. 2005; Cortez and Schultz-Cherry 2021). Therefore, we assessed the changes in the number of colonic goblet cells and the integrity of the colonic mucus layer in IAV-infected mice by PAS-specific staining. The present results are illustrated in Figure 3A. Mice in the H1N1 group had significantly reduced numbers of colonic goblet cells and the mucus coverage on their mucous membranes when compared to the negative control group. However, XEGMG treatment could restore the number of goblet cells to normal. The present results suggested that XEGMG stabilize the number of intestinal epithelial cells and effectively maintain the defense effect of the mucosal protective barrier.

IL-17A is a pro-inflammatory cytokine that plays an important role in host defense against microbial infection. IL-17A secretion can lead to a cascade of events involving neutrophil recruitment, inflammation and host defense. But excessive inflammation can lead to tissue damage. In addition, we determined the expression level of IL-17A in colon homogenates (Figure 3(B)). The results obtained demonstrated that, when compared with that in the negative control group, the expression of IL-17A in the H1N1 group was significantly increased (*p*** **<** **0.0001), while it was restored after treatment with XEGMG (*p*** **<** **0.0001). Since IL-17A is a signature cytokine produced by Th17 cells, this finding implies that XEGMG can possibly mediate immune responses to lung and intestinal diseases by regulating the Th17 cells.

### XEGMG maintains the gut microbial community of IAV-infected mice

**Figure 4.**
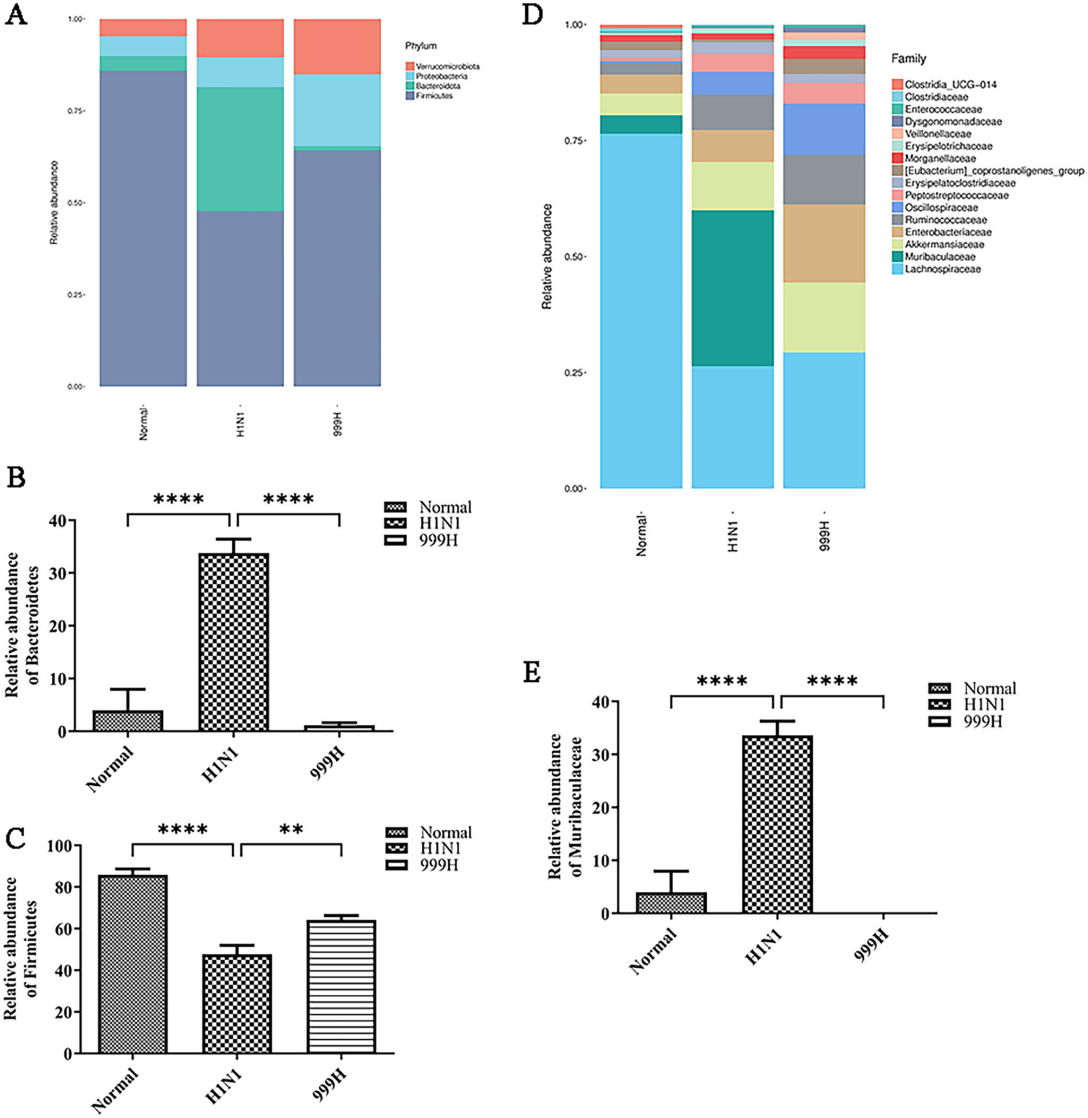
XEGMG regulated the dysbiosis of the gut microbial community of IAV-infected mice at day 4. (A) The relative abundance of bacteria at the phylum level. (B) The relative abundance of Firmicutes at the phylum level. (C) The relative abundance of Bacteroidetes at the phylum level. (D) The relative abundance of bacteria at the family level. (E) The relative abundance of Muribaculaceae at the family level. Data are presented as the mean ± standard error of the mean (*p < 0.05; **p < 0.01; ***p < 0.001; ****p < 0.0001 when compared with the H1N1 group (*n* = 4). H1N1, infected with A/FM/1/47(H1N1); N, mock-infected control group; 999 L, the infected mice were treated with 6 g/kg XEGMG intragastrically; 999H, treated with 12 g/kg XEGMG intragastrically; OST, treated with 22.75 mg/kg oseltamivir intragastrically.

The cumulative results of this study indicate that H1N1 infection can induce damage to the mouse colon, and the integrity of the intestinal flora is closely related to the function of the intestinal barrier (Sicard et al. 2017; Cai et al. 2020; Zhao and Maynard 2022). Accordingly, we analyzed the gut microbial community of mice. The results indicated that the intestinal bacterial phyla of the experimental mice are mainly composed of Verrucobacterium, Proteobacteria, Bacteroidetes, and Firmicutes, among others. Moreover, H1N1 infection indicated changes in the gut microbiota in the experimental mice (Figure. 4(A)). Quantitative analyses of several bacterial groups demonstrating significant changes indicated that the relative abundance of Firmicutes was significantly reduced (*p*** **<** **0.0001) and the abundance of Bacteroidetes was significantly increased (*p*** **<** **0.0001) in the H1N1 group relative to those in the negative control group. After high-dose XEGMG treatment, the relative abundance of Bacteroidetes was restored (*p*** **<** **0.0001), and the relative abundance of Firmicutes increased significantly (*p*** **<** **0.001) when compared with that of the H1N1 groups (Figures 4(B,C)). The abundance of the top 16 bacterial communities at the family level is presented in Figure 4(D). The abundance of Muribaculaceae was increased by H1N1 treatment (*p*** **<** **0.0001), while high-dose XEGMG decreased it (*p*** **<** **0.0001) (Figure 4(E)). These results demonstrated that XEGMG (12 g/kg) regulated the major flora such as Firmicutes, Bacteroidetes, and Muribaculaceae and played an important role in maintaining the gut microbial community of H1N1-infected mice.

## Discussion

The invasion and reproduction of viruses in the lungs can result in direct lung damage. Meanwhile, during H1N1 influenza virus infection, the viral RNA binds to the HA receptors in the respiratory epithelium, which activates the pattern-recognition receptors, resulting in the initiation of the cascade response of the innate and acquired immunity systems (Goraya et al. 2015; Chen et al. 2018; Zhang et al. 2020). These factors together caused pulmonary edema, damaged alveolar structure, and lead to massive infiltration of inflammatory cells and other symptoms of lung injury in influenza infection (Yoo et al. 2013). Intestinal immune damage caused by the influenza virus is a pathological link that has received much attention, and it is closely related to the prognosis of influenza. Our previous study demonstrated that influenza infection induces lung damage and indirect intestinal damage to the gut-lung axis by affecting the migration and differentiation of Th 17 cells, which can lead to a series of complications (Shi et al. 2020). On the other hand, the stabilization of the gut microbiota plays a positive role in exerting a protective effect on the lung barrier (Santo et al. 2021; Tang et al. 2021).

Although several anti-influenza virus drugs have been discovered and applied, drug resistance continues to emerge as the influenza virus continues to change abnormally, which reduces the effectiveness of the drugs. In addition, these anti-influenza drugs have various gastrointestinal adverse reactions. For example, the most common adverse reactions of oseltamivir are nausea and vomiting; zanamivir may cause bronchospasm; peramivir may cause diarrhea. The results of a genetic meta-analysis (Dobson et al. 2015) suggested that oseltamivir in adults with influenza accelerates time to clinical remission and reduces the risk of lower respiratory tract complications and hospital admissions, but increases the incidence of nausea and vomiting. Researchers are also concerned about whether the adverse reactions of these drugs outweigh their therapeutic effects. Therefore, TCM with milder efficacy is deemed a preferable treatment option for influenza infection in China.

XEGMG demonstrated the effect of clearing the heat and toxicity, which are often applied in the treatment of upper respiratory tract infections in children. TCM works synergistically through multiple active ingredients. It has also been confirmed by a large number of literature reports that most components of XEGMG exhibited anti-inflammatory, antibacterial, and antiviral activities, such as *Chrysanthemum morifolium* (Li et al. 2022), *Forsythia suspensa* (Gong et al. 2021), *Rehmannia glutinosa* (Li et al. 2022), and *Cynanchum atratum* (Zhang et al. 2022). In our study, we investigated the protective effect of XEGMG on viral pneumonia and intestinal injury in IAV-infected mice. Accordingly, we identified that high-dose XEGMG (12 g/kg) exhibited a protective effect on H1N1-induced lung injury at a 2LD_50_ infection dose, which can significantly reduce the lung index. The results of H&E staining from the lungs revealed that XEGMG could reduce pulmonary inflammatory cell infiltration. Furthermore, it was found that high-dose XEGMG could significantly reduce the expression of IL-1β and IL-6 in lung tissues, indicating that it has certain anti-inflammatory and antiviral activities *in vivo*. In addition, *Pogostemon cablin* (Chen et al. 2021), *Mentha canadensis* (Chumpitazi et al. 2018), and *Chrysanthemum morifolium* (Li et al. 2022) have been proven to exert protective effects on the gastrointestinal tract. Moreover, our results showed that XEGMG stabilizes the number of intestinal epithelial cells and effectively maintains the defense of the mucosal protective barrier. The results of intestinal H&E staining and PAS staining confirmed that H1N1 infection could induce intestinal barrier injury, as seen in inflammatory cell infiltration and intestinal goblet cell reduction in mice. However, the high-dose XEGMG group exhibited a certain protective effect on the intestinal mucosa, which was manifested as reduced infiltration of intestinal inflammatory cells and an increased number of goblet cells in the colon. All of these results together suggest that the active components of XEGMG can treat not only viral pneumonia but also gastrointestinal damage in children infected with influenza.

The composition and relative abundance of the gut bacteria can maintain the structure and immune homeostasis of the intestinal mucosa. The results of 16S rRNA gut bacteria sequencing in this study revealed that the mice in the model group had developed intestinal flora disturbances, wherein the relative abundance of Firmicutes was significantly decreased while those of Bacteroidetes and Muribaculaceae were significantly increased. Thus, high-dose XEGMG (12 g/kg) can restore this imbalance.

The role of gut-specific Th17 cells is critical for autoimmune diseases and body defense responses, as they recruit neutrophils and promote the secretion of antimicrobial factors in the bronchial epithelium (Littman and Rudensky 2010). Th17 mainly secretes pro-inflammatory factors, such as IL-17A and IL-22 (McAleer and Kolls 2014). IL-17A is a pro-inflammatory cytokine with the ability to recruit neutrophils. When extracellular pathogens invade the immune system, they promote a variety of cells to release inflammatory factors, which, in turn, amplify the inflammatory response (Iwakura et al. 2008). Past studies have demonstrated that IL-17A produced by Th17 cells in the intestinal mucosa can disrupt the intestinal barrier and promote systemic inflammation, inhibit the clearance of viral particles, and synergize with the innate immune system to exacerbate lung inflammation (Bermejo-Martin et al. 2009; Wang et al. 2011). Significant changes in the expression of IL-17A were detected in this study, implying that XEGMG could reduce the lung and intestinal injury caused by influenza in mice by inhibiting the increase of IL-17A levels.

In our study, XEGMG not only demonstrated mild efficacy but also effectively improved intestinal damage, thereby confirming the advantages of TCM for multi-organ protection and providing evidence for the safety of clinical medication for children. In the follow-up study, we plan to further explore the protective effect of XEGMG on the gastrointestinal tract and its impact on the intestinal flora. Furthermore, we detected changes in the expression of IL-17A in the colon in this study. Accordingly, the verification of whether this is related to the balance of Th17/Treg cells warrants further experimentation.

## Conclusions

The findings of this study clarified the protective effects of XEGMG on viral pneumonia as well as its protective effect on the intestinal tract and the remodeling of the intestinal flora. XEGMG has been used as an OTC drug for treating upper respiratory tract disorders. Our findings extend its application to lower respiratory tract infections, thereby validating its protective effect on mild pneumonia and introducing its protective effect against intestinal damage. Overall, our study provides a certain experimental basis for the clinical efficacy and highlights the characteristics of the Chinese medicine compound XEGMG.

## Data Availability

The data used to support the findings of this study are included in the article.
